# A meta-analysis of comparison of proximal gastrectomy with double-tract reconstruction and total gastrectomy for proximal early gastric cancer

**DOI:** 10.1186/s12893-019-0584-7

**Published:** 2019-08-22

**Authors:** Shengnan Li, Lihu Gu, Zefeng Shen, Danyi Mao, Parikshit A. Khadaroo, Hui Su

**Affiliations:** 1 The Second Clinical Medical College, Zhejiang Chinese Medical University, Zhejiang, Hangzhou China; 2 Department of General Surgery, HwaMei Hospital, University of Chinese Academy of Sciences, Northwest Street 41, Haishu District, Ningbo, Zhejiang, 315010 China; 3 Basic Medical College, Zhejiang Chinese Medical University, Zhejiang, Hangzhou China; 4 Monash University School of Public Health and Preventive Medicine, Melbourne, Australia; 5 Department of General Surgery, The first Affiliated Hospital of Jinan University, Huangpu Road West 613, Tianhe District, Guangzhou, 510630 Guangdong China

**Keywords:** Proximal gastrectomy, Double-tract, Total gastrectomy, Early gastric cancer, Meta-analysis

## Abstract

**Background:**

In theory, proximal gastrectomy with double-tract reconstruction (PG-DT) was superior to total gastrectomy (TG) in hematologic and nutritional outcomes. However, its clinical effects in proximal early gastric cancer (EGC) have been controversial.

**Methods:**

The purpose of this study was to investigate the outcomes of laparoscopic proximal gastrectomy with double-tract reconstruction (LPG-DT) for proximal EGC. For this systematic review and meta-analysis, we searched for articles published before December of 2018 in the following databases: PubMed, Web of Science, EBSCO, Medline, and Cochrane Library.

**Results:**

The results showed no significant difference in the anastomotic stenosis (OR = 0.91, 95%CI = 0.33–2.50, *p* = 0.85) and reflux esophagitis (OR = 1.87, 95%CI = 0.62–5.65, *p* = 0.27) between LPG-DT and laparoscopic total gastrectomy (LTG). The vitamin B12 supplementation rate in the LPG-DT group was lower than the LTG group (OR = 0.06, 95%Cl = 0.01–0.59, *p* = 0.02).

**Conclusions:**

Due to comparable clinical effect, PG-DT is comparable to TG for patients with proximal EGC. In addition, LPG-DT not only appears superior to TG in terms of preventing vitamin B12 deficiency, but also does not increase the risk of anastomotic stricture and reflux esophagitis.

**Electronic supplementary material:**

The online version of this article (10.1186/s12893-019-0584-7) contains supplementary material, which is available to authorized users.

## Introduction

Recently, with the prevalence of endoscopic techniques, the incidence of proximal gastric cancer (lesion located in the upper third of the stomach), especially proximal early gastric cancer (EGC) has been increasing in worldwide [1]. So far, for patients with proximal EGC who have lesions unsuitable for endoscopic treatment, total gastrectomy (TG) is the most major surgical procedure for the radical treatment. However, TG has many potential disadvantages, especially in hematological and nutritional status [2]. Thus, some researchers have suggested the proximal gastrectomy (PG) as an alternative to TG for proximal EGC [3]. Unfortunately, severe reflux esophagitis and anastomotic stenosis are the major factors that limit the clinical application of this surgery. Besides, the operation procedure needs to be further standardized [4, 5].

Some scholars suggested that patients with proximal EGC could be treated with proximal gastrectomy with double-tract reconstruction (PG-DT), to avoid anastomotic complications [6, 7]. It was reported that the incidences of reflux symptoms, usage of proton pump inhibitors, and anastomotic strictures were significantly lower in the double-tract anastomosis group as compared to the esophagogastrostomy group [6]. However, a recent multicenter study from the western experience showed that patients who underwent PG have an increased mortality rate and a higher risk of reflux esophagitis and anastomotic stricture [8].

In addition, in view of the prognosis of EGC is excellent, the improvement of postoperative quality of life and the safe application of minimally invasive surgery have become the research hotspot in recent years. There is no doubt that laparoscopic proximal gastrectomy (LPG) is one of the most promising treatments for patients with proximal EGC [9]. But LPG has remained controversial mainly due to a lack of evidence from large-scale studies. And there were few meta-analyses about this problem in comparison of laparoscopic proximal gastrectomy with double-tract reconstruction (LPG-DT) and laparoscopic total gastrectomy (LTG) for treatment effects. So, this meta-analysis is a comparison of the short-term and long-term outcomes of LPG-DT and LTG for proximal EGC through comprehensive retrieval and pooled analysis.

## Methods

In the electronic databases of PubMed, Web of science, EBSCO and the Cochrane library, a comprehensive literature search strategy was performed by retrieving the keywords “proximal gastrectomy” and “total gastrectomy” until December 2018. To avoid omitting any potential studies, we manually reviewed the references of included literature. Non-English studies will be excluded. Institutional review board approval of our hospital was obtained for this study.

### Study selection

Two authors individually conducted the search and independently reviewed and extracted data from each study. The search results were compared, and any disagreement in opinions were resolved by further discussion. This meta-analysis included studies that met the following criteria: (1) studies focusing on patients with early gastric cancer; (2) comparative studies between PG-DT and TG; (3) having reported detailed/available data of the surgical results, including short- and/or long-term outcomes. But these studies were excluded if they were (1) non-original articles; (2) not comparing PG-DT and TG; (3) not relevant outcome or detailed data.

### Data extraction

Data extracted included study characteristics (such as author, region, study period, design, case number), patient demographics (such as age, gender, body mass index), tumor stage, surgical results (such as operation time, the number of retrieved lymph nodes, perioperative complication and resection margin), long-term outcomes (hematological and nutritional outcomes), and oncological outcome. Corresponding authors were contacted if further information was needed.

### Evaluation of quality of the studies

This meta-analysis was conducted on the recommendation of guidelines of the Preferred Reporting Items for Systematic Review and Meta-Analysis (PRISMA) 2009 Checklist (Additional file 1: Table S1) [10]. Two authors independently assessed the quality of the included researches, according to the Newcastle-Ottawa Quality Assessment Scale (NOS) checklist, which consisted of eight items, divided into three aspects (selection, comparability and outcome) with a maximum number of 9 stars [11].

### Statistical methods

RevMan 5.3.5 software for Windows® was used to analyze the data. According to data characteristics, dichotomous variables and continuous variables were pooled analyzed using estimation of odds ratios (OR) and weighted mean difference (WMD) with a 95% Confidence interval (CI), respectively. Heterogeneity across studies was evaluated by Cochrane Q-test and *p*-values. We considered heterogeneity to be present if the *p* < 0.1. The fixed-effect model was used for meta-analysis in cases of nonsignificant heterogeneity. On the contrary, if there was a significant heterogeneity, the random-effect model was used. Publication bias was evaluated by Egger’s test. Egger’s test was not suitable for subgroup analysis, if less than ten studies were included due to low sensitivity of qualitative and quantitative tests. All statistical tests were performed two-sided, and *p* < 0.05 was considered statistically significant.

## Results

### Literature search

**Fig. 1:**
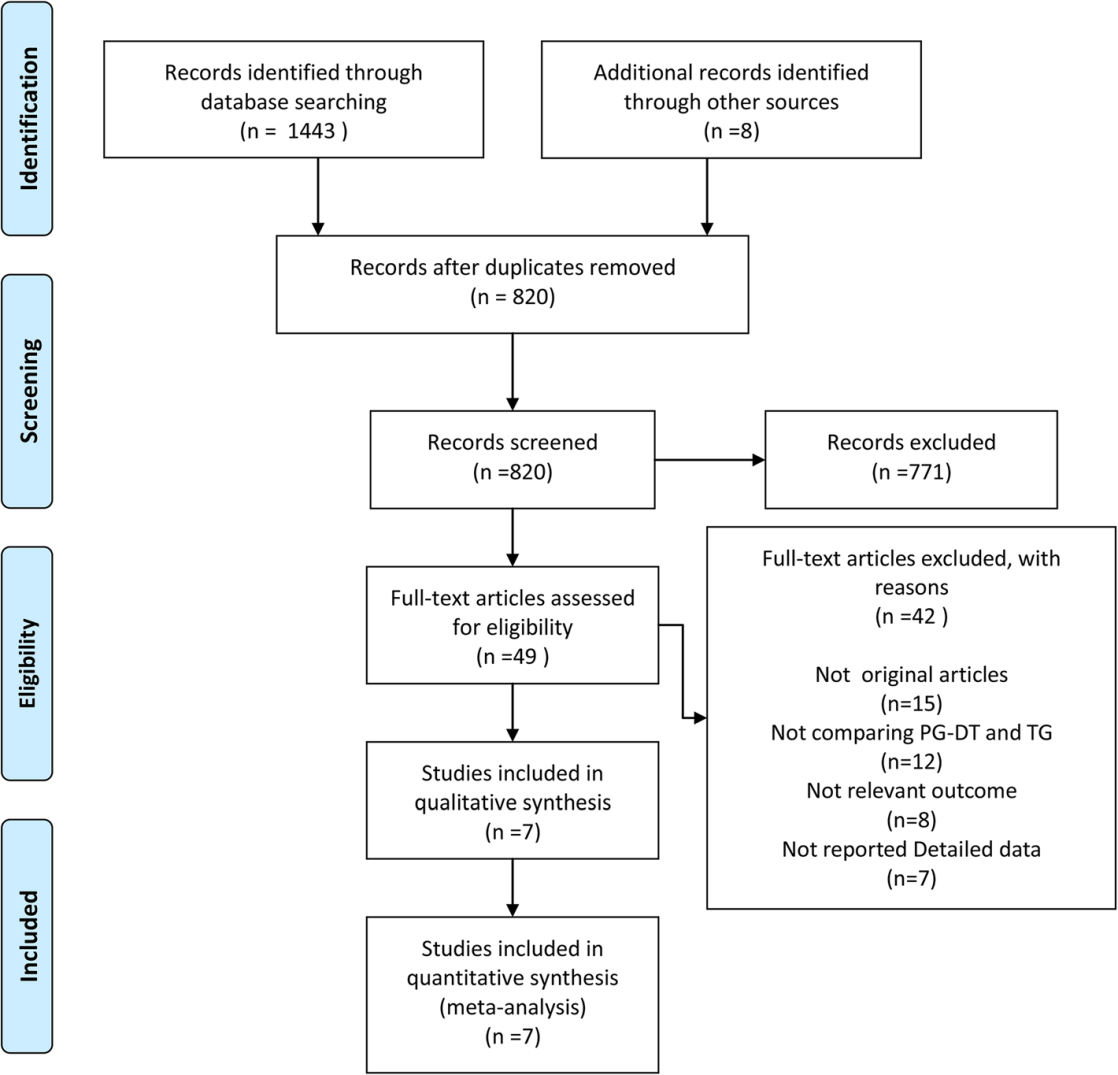
Flow chart of study selection

A total of 1451 studies were identified after the initial search. After deletion of 631 duplicates, 771 studies were additionally excluded by careful review of the title and/or abstract. After screening the title and abstract, 49 studies were evaluated via full-text articles. At last, seven articles [7, 12–17] published were included for quantitative synthesis from 2016 to 2018. The flow diagram were summarized is shown in Fig. 1.

### Study characteristics

Based on the inclusion and exclusion criteria, seven retrospective studies were included for this review. The characteristics of each study were shown in Table 1. The seven studies included a total of 592 patients: PG-DT was performed in 347 patients and TG was performed in 245 patients. The sample size of each study varied from 30 to 248 patients. All patients received laparoscopic or robotic surgery, of which 533 cases (90.0%) of belonged to stage I proximal gastric cancer. In the PG-DT group, 331 patients (95.4%) were stage I proximal gastric cancer, and in the TG group, 202 cases (82.4%) were stage I proximal gastric cancer.

**Table 1:** Characteristics of studies included in the meta-analysis

Author, year	Country	Surgical procedures		Patients, n		Follow-up (months)		Oncological outcomes		NOS
		PG-DT	TG	PG-DT	TG	PG-DT	TG	PG-DT	TG	
Cho et al*,* 2018	Korea	Laparoscopic /Robotic	Laparoscopic /Robotic	38	42	24		100%	100%	8
Furukawa et al*,* 2018	Japan	Laparoscopic	Laparoscopic	27	48	30	48.5	100%	100%	8
Jung et al, 2017	Korea	Laparoscopic	Laparoscopic	92	156	12–24		100%	98%	9
Kim et al*,* 2016	Korea	Laparoscopic	Laparoscopic	17	17	18		100%	100%	8
Nomura et al*,* 2018	Japan	Laparoscopic	Laparoscopic	15	30	12		100%	100%	7
Park et al*,* 2018	Korea	Laparoscopic	Laparoscopic	46	34	24		100%	98%	8
Sugiyama et al, 2018	Japan	Laparoscopic	Laparoscopic	10	20	6–12		100%	100%	8

*PG-DT*, Proximal gastrectomy with double tract reconstruction; *TG*, Total gastrectomy;

*NOS*, Quality Assessment based on Newcastle-Ottawa Scale

### Patient’s clinicopathologic features

In the analysis of patients’ basic conditions, there were differences in gender (OR = 1.89,95% CI = 1.26–2.84, *p* = 0.002, fixed-effects model) and American Society of Anesthesiologist (ASA) (OR = 0.43, 95%CI = 0.22–0.83, *p* = 0.01, fixed-effects model) between both groups of patients underwent surgical treatment (LPG-DT vs. LTG). In terms of tumor lesion, patients with LPG-DT had smaller tumor sizes than those with LTG (WMD = -0.94, 95%CI = -1.26-(− 0.62), *p* < 0.001, fixed-effects model). The T stage (OR = 2.21, 95% CI = 1.17–4.17, *p* = 0.01, fixed-effects model) and tumor stage (OR = 2.70, 95%CI = 1.35–5.39, *p* = 0.005, fixed-effects model) were earlier in the LPG-DT group. However, there was no significant difference in N Stage between both groups (OR = 0.77, 95%CI = 0.34–1.71, *p* = 0.52, fixed-effects model) (Table 2).

**Table 2:** Subgroup analysis of comparison between LPG-DT and LTG

	No. of studies	OR/WMD	95%CI	*p*	Heterogeneity		Effect model
					I^2^	*p*	
Age	6	0.18	−3.07-3.42	0.92	69%	0.007	Random
Gender (male)	7	1.89	1.26–2.84	0.002	0%	0.50	Fixed
ASA (I)	3	0.43	0.22–0.83	0.01	1%	0.36	Fixed
BMI (kg/m^2^)	5	−0.40	−1.76-0.96	0.57	86%	< 0.001	Random
Tumor size (cm)	4	−0.94	−1.26-(−0.62)	< 0.001	0%	0.59	Fixed
T-stage (I)	4	2.21	1.17–4.17	0.01	29%	0.24	Fixed
N-Stage (N+)	4	0.77	0.34–1.71	0.52	0%	0.53	Fxied
Stage (I)	6	2.70	1.35–5.39	0.005	0%	0.61	Fixed
Operation time (min)	6	−10.43	−25.64-4.77	0.18	69%	0.007	Random
Blood loss (ml)	4	3.74	−57.37-64.84	0.90	88%	< 0.001	Random
Proximal resection margin (cm)	4	−0.97	−1.80-(−0.14)	0.02	77%	0.004	Random
Distal resection margin (cm)	4	−8.30	−9.57-(−7.03)	< 0.001	76%	0.006	Random
No. of retrieved LNs	5	−11.28	−13.52-(−9.04)	< 0.001	26%	0.25	Fixed
Length of hospital stay (day)	6	−0.21	−1.21-0.80	0.68	10%	0.35	Fixed
C-D grade I or more	4	0.87	0.36–2.13	0.76	65%	0.04	Random
C-D grade II or more	4	0.73	0.44–1.21	0.22	0%	0.46	Fixed
C-D grade III or more	3	0.35	0.12–1.07	0.07	51%	0.15	Fixed
Cholecystitis	4	1.41	0.44–4.52	0.56	0%	0.64	Fixed
Fluid collection	3	0.74	0.30–1.86	0.53	45%	0.16	Fxied
Leakage	6	0.81	0.34–1.94	0.64	0%	0.68	Fixed

*LPG-DT*, Laparoscopic proximal gastrectomy with double-tract reconstruction; *LTG*, Laparoscopic total gastrectomy; *OR*, Odds ratios; *WMD*, Weighted mean difference; 95%CI, 95% Confidence interval; *ASA*, American Society of Anesthesiologist; *BMI*, Body mass index; *LNs*, Lymph nodes; *C-D*, Clavien-Dindo

### Surgical conditions and short-term outcomes

In terms of surgical conditions, patients in the LTG group had longer proximal (WMD = -0.97, 95%CI = -1.80-(− 0.14), *p* = 0.02, random-effects model) and distal resection margins (WMD = -8.30, 95%CI = -9.57-(− 7.03), *p* < 0.001, random-effects model) and more retrieved lymph nodes (WMD = -11.28, 95%CI = -13.52-(− 9.04), *p* < 0.001, fixed-effects model). However, there was no significant difference in operative time (WMD = -10.43, 95%CI = -25.64–4.77, *p* = 0.18, random-effects model) and intraoperative blood loss (WMD = 3.74, 95%CI = − 57.37-64.84, *p* = 0.90, random-effects model) between both groups (Table 2).

Besides, there was no significant difference in short-term outcomes between both groups, including postoperative the length of hospital stay (WMD = -0.21, 95%CI = -1.21–0.80, *p* = 0.68, fixed-effects model), all complications (Clavien-Dindo Grade I or more) (OR = 0.87, 95%CI = 0.36–2.13, *p* = 0.76, random-effects model), severe complications (Clavien-Dindo Grade III or more) (OR = 0.35, 95%CI = 0.12–1.07, *p* = 0.07, fixed-effects model), and anastomotic leakage (OR = 0.81, 95%CI = 0.34–1.94, *p* = 0.64, fixed-effects model) (Table 2).

### Long-term outcomes

**Fig. 2:**
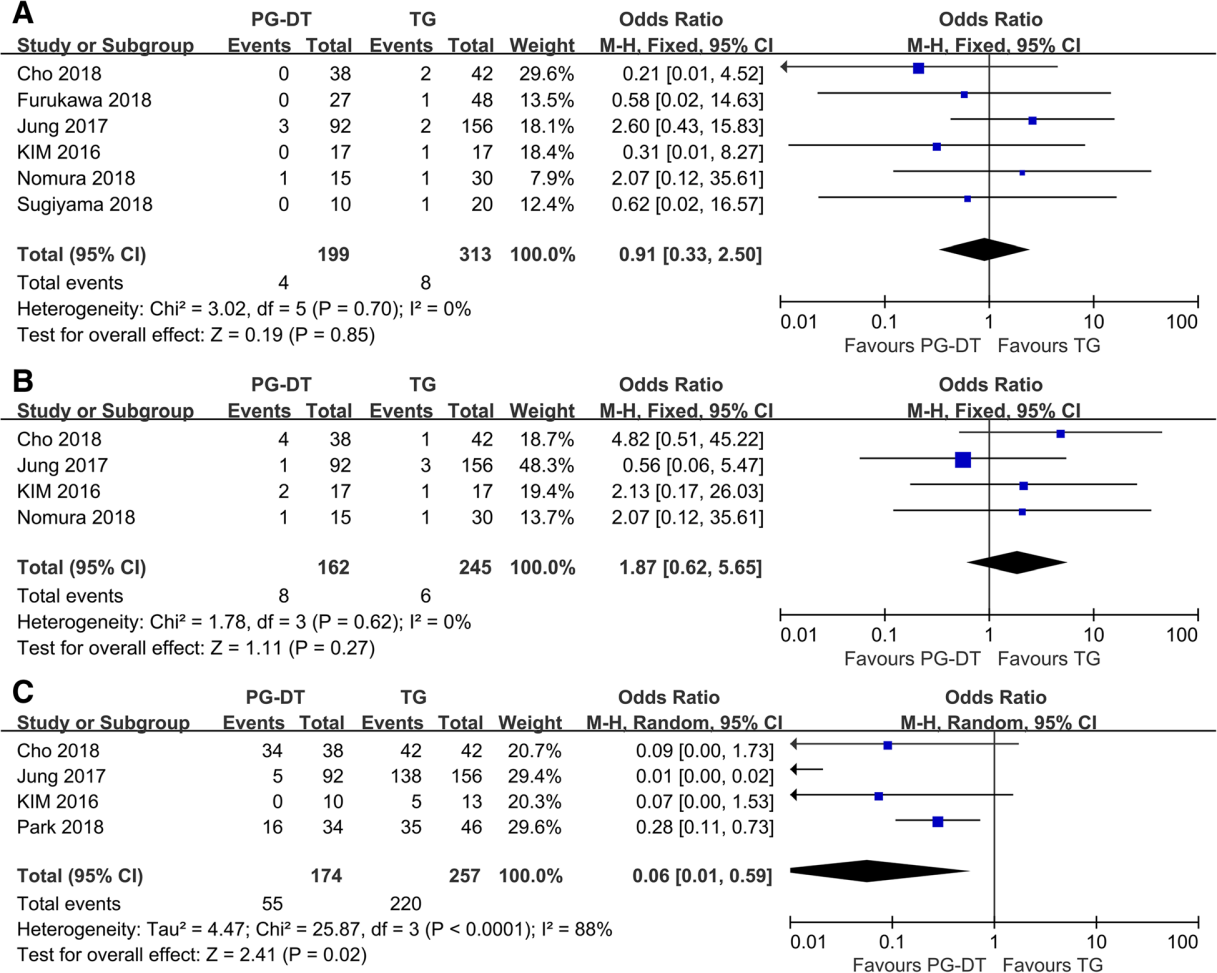
Forest plot describing the association between surgery and long-term outcomes of patients with proximal EGC. (A) anastomotic stricture, (B) reflux esophagitis, (C) vitamin B_12_ deficiency rate

The long-term results including anastomotic stenosis, reflux, hematological status, vitamin B_12_, and oncological outcomes were analyzed. There was also no statistically significant difference between anastomotic stenosis (OR = 0.91, 95%CI = 0.33–2.50, *p* = 0.85, fixed-effects model) and reflux (OR = 1.87, 95%CI = 0.62–5.65, *p* = 0.27, fixed-effects model) (Fig. 2). By comparing the hematological and nutritional outcomes between both groups, body mass index (BMI) was mentioned in five studies, among which three showed statistical difference and two showed no statistical difference. Hemoglobin was investigated in six studies, among which only one showed statistical difference, while the other five showed no statistical difference. Ferritin was mentioned in two studies, they all showed no statistical difference. In addition, there were many studies investigated total protein, albumin, total cholesterol and total lymphocyte count, none of the results were statistically significant (Additional file 2: Table S2). Four of these studies reported postoperative vitamin B_12_. The vitamin B_12_ supplementation rate in the LPG-DT group was lower than the LTG group (OR = 0.06, 95%Cl = 0.01–0.59, *p* = 0.02, random-effects model) (Fig. 2). At the end of follow-up, no patients underwent LPG-DT had recurrence or death. In the LTG group, a total of 4 patients relapsed.

## Discussion

PG is theoretically the ideal treatment option for proximal EGC [18]. However, the risk of reflux esophagitis and anastomotic stenosis is a huge challenge for the widespread use of PG. To improvement these disadvantages, several modified reconstruction procedures, including jejunal interposition, jejunal pounch interposition, double-flap, and double-tract anastomosis, have been attempted [6, 19–21]. According to previous studies, PG-DT is one of the most promising procedures, in reducing anastomotic complications (reflux esophagitis and anastomotic stenosis) and improving operative quality of life [7, 12]. Thus, the purpose of this meta-analysis is to compare the safety and feasibility of LPG-DT with LTG for patients with clinical stage I proximal gastric cancer.

In this study, the tumor size of the LTG group was significantly larger than that of the LPG-DT group. Although tumor depth and tumor staging was significantly earlier in the LPG-DT group than in the LTG group, there was no difference in lymph node metastasis. It seems that the size and depth of the tumor are factors in the choice of surgical procedure. Masuzawa et al. [22] reported that tumor size and histology could influence the size of the remnant stomach, which was an important factor in choosing the procedure. So, we believed that LPG-DT was appropriate for proximal EGC, and the tumor size was small. In terms of operation time and blood loss, no significant difference was found between the two groups. Thus, LPG-DT is not a complicated procedure, compared LTG. Although resection margins were shorter for LPG-DT, intraoperative frozen biopsies confirm disease-free resection margins (R0 resection). In other words, PG can maximize the retention of gastric stump. In addition, consistent with previous findings [20, 23], this study showed that fewer lymph nodes were retrieved every patient in the LPG-DT group because of different lymph node metastasis dissection extent. The TNM staging system recommends that no less than 16 lymph nodes should be resected for radical gastrectomy [3]. And several studies have shown that the number of lymph nodes retrieved is closely associated with postoperative pathologic staging and prognostic assessment [24, 25]. Gholami et al. [26]. demonstrated that dissection of 16 or more lymph nodes was associated with better survival in all patients. However, some studies suggested that dissection of more than 16 LNs did not show improvement in the prognosis in stage I patients [27, 28]. In our previous study [29], there was a low incidence of lymph node metastasis in EGC. So, we believed that LPG-DT with less than D2 lymph node dissection appears to be reasonable.

An et al. [30] and Rosa et al. [8] reported that PG with esophagogastrostomy resulted in a higher complication rate than TG and that PG with esophagogastrostomy led to a higher frequency of stenosis and reflux than did TG. Nishigori et al. [23] also demonstrated that PG with esophagogastrostomy had a higher probability of anastomotic stenosis. There was no significant difference in the short-term outcomes between the two groups (LPG-DT versus LTG) in this article. Whether in all complications or severe complications, the present study showed that LPG-DT was a feasible and safe technique. Most importantly, the results of this study showed that LPG-DT did not increase the incidences of reflux esophagitis and anastomotic stenosis, compared LTG. Besides, Aburatani et al. [6] suggested that the incidence of both reflux esophagitis and anastomotic stenosis was lower in the PG-DT group than in the PG with esophagogastrostomy group. We could conclude that LPG-DT was a good alternative to LTG, because of similar complication rate.

There is no doubt that the long-term quality of life is one of the important factors to evaluate the value of surgery, especially for patients with EGC. Several previous studies had reported hematological and nutritional outcomes in patients underwent PG. Cho et al. [12] reported that postoperative hematologic indexes, including hemoglobin, ferritin, transferrin saturation, and anemia, showed no significant difference between minimally invasive PG-DT and minimally invasive TG. Protein, albumin, cholesterol, and other nutritional parameters were also comparable between the two groups. They also concluded that although the proportion of patients who requiring vitamin B_12_ supplements was smaller in the PG-DT group, the cumulative incidence of vitamin B_12_ deficiency after PG-DT was similar to that after TG. Jung et al. [14] thought that the change rate of body weight in LPG-DT group was significantly lower than in LTG group. The serum vitamin B_12_ level in the LPG-DT group was significantly higher than in the LTG group. Research by Kim and his colleagues [15] observed that LPG-DT was beneficial with regards to the absorption of iron and vitamin B_12_ compared to LTG. A recent study has shown that body weight and skeletal muscle index reduction rates were lower in the LPG-DT group than in the LTG group [17].

In this present study, a significantly smaller proportion of the LPG-DT patients required vitamin B_12_ supplementation compared to the LTG group. PG-DT preserves the gastric antrum and part of the gastric body, allowing for food reserve, which indicates that these areas of the stomach are potential sources of intrinsic factors. TG is known to have a risk of leading to vitamin B_12_ deficiency compared to distal gastrectomy [31]. Because the main source of intrinsic factors in the stomach is parietal cells, which are mainly located in the body and fundus, the ability of PG to prevent vitamin B_12_ deficiency is questionable. Our analysis showed that retention of the distal stomach assists in the absorption of the vitamin B_12_, possibly due to retention of a partial gastric body.

This systematic review and meta-analysis incorporated seven articles that compared the long-term hematological and nutritional indicators, including postoperative BMI, hemoglobin, ferritin, total protein, albumin, to patients with LPG-DT and LTG. Unfortunately, the analysis of forest plots with related indicators could not be obtained due to the inability to obtain the original data. However, no study showed that the hematological and nutritional status of patients who underwent LPG-DT was worse than that of patients underwent LTG.

Finally, in terms of oncology prognosis, the two groups had similar outcomes. Yoo et al. [4] reported that PG might increase the likelihood of local recurrence, although it would not affect long-terms in terms of survival and mortality. But the study included many patients with advanced gastric cancer. In this study, almost all the patients included had EGC. There was no recurrence or death in the patients with confirmed stage I proximal gastric cancer after LPG-DT. Therefore, we believed that LPG did not negatively influence the prognosis compared to LTG in EGC.

To our knowledge, this is the first meta-analysis investigates the short-term and long-term results of LPG-DT versus LTG. Some limitations of this study need to be emphasized. All included articles were the small sample retrospective studies, which inevitably led to a decline in the level of evidence in this paper. No prospective studies or clinical trials were found through systematic and comprehensive retrieval. Meanwhile, Egger’s test of publication bias was not performed on this analysis due to the insufficient number of included studies. Finally, the participants were all from Asian countries with high incidence of gastric cancer (South Korea and Japan). The conclusions of this study cannot be directly applied to other countries. Thus, a prospective randomized trial that compare LPG-DT with LTG should be performed to confirm these observations.

## Conclusion

This review showed that due to comparable clinical effect, LPG-DT is comparable to LTG for patients with proximal EGC. In addition, LPG-DT not only appears superior in preventing vitamin B_12_ deficiency, but also does not increase a risk of anastomotic stricture and reflux esophagitis compared to LTG.

## Additional files

Additional file 1: **Table S1.** PRISMA 2009 checklist. (DOCX 17 kb)

Additional file 2: **Table S2.** Comparison of hematological and nutritional outcomes between both groups. (DOCX 25 kb)

## Data Availability

The datasets supporting the conclusions of this article are included within the article and its additional files.

## Abbreviations

ASA: American Society of Anesthesiologist
CI: Confidence interval
EGC: early gastric cancer
LPG: laparoscopic proximal gastrectomy
LPG-DT: laparoscopic proximal gastrectomy with double-tract reconstruction
LTG: laparoscopic total gastrectomy
NOS: Newcastle-Ottawa Quality Assessment Scale
OR: odds ratios
PG: proximal gastrectomy
PG-DT: proximal gastrectomy with double-tract reconstruction
PRISMA-P: Preferred Reporting Items for Systematic Review and Meta-Analysis Protocols
TG: total gastrectomy
WMD: weighted mean difference
