# Supplementary material for: A meta-analysis of comparison of proximal gastrectomy with double-tract reconstruction and total gastrectomy for proximal early gastric cancer
Source: BMC Surg. 2019 Aug 22;19:117. doi: 10.1186/s12893-019-0584-7 (PMC6704512; doi:10.1186/s12893-019-0584-7)
Supplement: Supplementary file 2 — Table S2. Comparison of hematological and nutritional outcomes between both groups. (DOCX 25 kb) [file 12893_2019_584_MOESM2_ESM.docx]

| Author | BMI | Hb | Ferritin | Iron | Total protein | Albumin | Total cholesterol | Total lymphocyte count |
| --- | --- | --- | --- | --- | --- | --- | --- | --- |
| Cho *et al* | 0.591 | 0.250 | 0.602 | **0.007** | 0.678 | 0.743 | 0.144 | 0.938 |
| Furukawa *et al* | >0.05 | >0.05 | NA | NA | NA | >0.05 | NA | NA |
| Jung *et al* | **0.049** | **0.002** | NA | NA | >0.05 | >0.05 | >0.05 | NA |
| Kim *et al* | NA | >0.05 | >0.05 | >0.05 | >0.05 | >0.05 | NA | NA |
| Nomura *et al* | **0.024** | NA | NA | NA | NA | NA | NA | NA |
| Park *et al* | NA | 0.495 | NA | 0.293 | 0.357 | 0.615 | 0.768 | NA |
| Sugiyama *et al* | **0.004** | 0.177 | NA | NA | NA | 0.470 | NA | 0.772 |

**Additional file 2: Table S2.** Comparison of hematological and nutritional outcomes between both groups.

NA, not available; BMI, Body mass index; Hb, Hemoglobin
